# The Ecology of Human Sleep (EcoSleep) Cohort Study: Protocol for a longitudinal repeated measurement burst design study to assess the relationship between sleep determinants and outcomes under real‐world conditions across time of year

**DOI:** 10.1111/jsr.14225

**Published:** 2024-07-22

**Authors:** Anna M. Biller, Nayab Fatima, Chrysanth Hamberger, Laura Hainke, Verena Plankl, Amna Nadeem, Achim Kramer, Martin Hecht, Manuel Spitschan

**Affiliations:** ^1^ Department Health and Sport Sciences, Chronobiology and Health Technical University of Munich, TUM School of Medicine and Health Munich Germany; ^2^ Max Planck Institute for Biological Cybernetics Research Group Translational Sensory and Circadian Neuroscience Tübingen Germany; ^3^ Department of Psychiatry and Psychotherapy Technical University of Munich, TUM School of Medicine and Health Munich Germany; ^4^ Department of Psychology Ludwig Maximilian University Munich Germany; ^5^ Laboratory of Chronobiology Charité‐Universitätsmedizin Berlin Berlin Germany; ^6^ Department of Psychology Helmut Schmidt University Hamburg Germany; ^7^ TUM Institute for Advanced Study (TUM‐IAS) Technical University of Munich Garching Germany

**Keywords:** individual sleep differences, naturalistic conditions, photoperiod, prospective cohort study, season, sleep architecture, sleep variability

## Abstract

The interplay of daily life factors, including mood, physical activity, or light exposure, influences sleep architecture and quality. Laboratory‐based studies often isolate these determinants to establish causality, thereby sacrificing ecological validity. Furthermore, little is known about time‐of‐year changes in sleep and circadian‐related variables at high resolution, including the magnitude of individual change across time of year under real‐world conditions. The Ecology of Human Sleep (EcoSleep) cohort study will investigate the combined impact of sleep determinants on individuals’ daily sleep episodes to elucidate which waking events modify sleep patterns. A second goal is to describe high‐resolution individual sleep and circadian‐related changes across the year to understand intra‐ and inter‐individual variability. This study is a prospective cohort study with a measurement‐burst design. Healthy adults aged 18–35 years (*N* = 12) will be enrolled for 12 months. Participants will continuously wear actimeters and pendant‐attached light loggers. A subgroup will also measure interstitial fluid glucose levels (six paticipants). Every 4 weeks, all participants will undergo three consecutive measurement days of four ecological momentary assessments each day (‘bursts’) to sample sleep determinants during wake. Participants will also continuously wear temperature loggers (iButtons) during the bursts. Body weight will be captured before and after the bursts in the laboratory. The bursts will be separated by two at‐home electroencephalogram recordings each night. Circadian phase and amplitude will be estimated during the bursts from hair follicles, and habitual melatonin onset will be derived through saliva sampling. Environmental parameters (bedroom temperature, humidity, and air pressure) will be recorded continuously.

## 1 INTRODUCTION

Human sleep is influenced by biological, behavioural, environmental, and personal and socioeconomic factors (Philippens et al., 2022). The impact of these *sleep determinants* on sleep quantity and quality is subject to inter‐individual differences (Philippens et al., 2022). In contrast to the ‘real world’, it is possible to isolate specific sleep determinants to study them in‐depth under well‐controlled laboratory conditions to determine causality by sacrificing ecological validity. To understand human sleep *in situ*, it is crucial to study how these determinants *in combination,* instead of *in isolation,* contribute to individual sleep episodes.

Current sleep hygiene recommendations are mostly generic and only sometimes prove successful in practice because they are misunderstood, applied wrongly or do not target the individual needs and situations of the person (Irish et al., 2015; Philippens et al., 2022; Voinescu & Szentagotai‐Tatar, 2015). As healthy sleep and entrained circadian rhythms are essential for maintaining health to prevent disease (Foster & Wulff, 2005; Garbarino et al., 2021; Luyster et al., 2012), identifying individual determinants and their respective weight is crucial for informing future personalised prevention programmes and interventions (Philippens et al., 2022). The more we understand how various factors and responses are connected, the better we can target unique individual needs (Lillie et al., 2011; Philippens et al., 2022). To achieve this goal, more knowledge about the day‐to‐day variability of sleep and circadian rhythms in an individual is needed.

Sleep also changes throughout the year. As exposure to light not only shapes sleep architecture, entrains circadian rhythms and influences melatonin production (Blume et al., 2019; Cajochen et al., 2022; Chellappa et al., 2013; Duffy & Wright, 2005; Wams et al., 2017), the change in relative abundance and timing of light across the year in locations further away from the equator could have an impact on sleep and circadian‐related variables. However, evidence on seasonal effects is very mixed and unclear (as summarised by Mattingly et al. [2021] on sleep duration and timing). Only a handful of laboratory‐based studies with small sample sizes have explicitly assessed seasonal changes in sleep duration and timing and even less on architecture.

For example, Wehr (1991) showed prolonged sleep and melatonin secretion under controlled shorter photoperiods in eight healthy participants. Yet in a later study, no difference in sleep duration or melatonin secretion was found between summer and winter (*N* = 21) despite differences in self‐recorded light exposure (Wehr et al., 1995). Van Dongen et al. (1997) assessed sleep electroencephalogram (EEG) and rectal temperature once per month over one year in six healthy participants under controlled temperature conditions. The phase of temperature and slow‐wave sleep (SWS) onset were ~45 min earlier in summer than winter and a general trend of later SWS in winter was overserved. Honma et al. (1992) studied ten male participants in conditioned laboratories for four days per season and showed phase delays of rectal temperature, plasma melatonin and sleep episode of 83–95 min in winter compared to summer. No difference in sleep duration but earlier sleep onset and offset were found in winter. In a cross‐sectional study in a sleep clinic by Askenasy and Goldstein (1995), rapid eye movement (REM) sleep duration and percentage were higher and REM latency was shorter in winter/spring compared to summer/autumn (*N* = 615), a finding which could not be replicated by Herer and Lavie (1997) who studied male patients with sleep apnea (*N* = 706). In patients with disturbed sleep (*N* = 292), Seidler et al. (2023) found longer total sleep time (TST) and REM‐sleep duration during winter than summer/spring, longer REM‐sleep latency during autumn than spring, and stable SWS except for a marked drop in autumn. However, these findings might look different in longitudinal studies with healthy participants.

Outside of the laboratory, there is also mixed evidence on the influence or association of season, time of year or photoperiod on sleep (Ferguson et al., 2021; Mattingly et al., 2021). This may be due to differences in study designs, data sets (either assessed objectively with actimetry or wearables, self‐reported through diaries, or large‐scale mobile application data or phone usage data), small effect sizes, and consideration of potential moderating variables (e.g., actual temperature and weather data at time of assessment instead of only the categorical variable *season*) (Mattingly et al., 2021). Another limitation is that many studies compare only a few data points sampled per season (an exception is the laboratory‐based study by Van Dongen et al. [1997], or actimetry/wearable studies such as Mattingly et al. [2021] with continuous wearable monitoring over 12 months). Self‐reported data and evidence from data repositories have larger sample sizes, and tend to support the existence of seasonal differences in sleep for specific groups but they often lack data on sleep timing (Mattingly et al., 2021). Clear influences or associations of season and sleep timing and duration are thus mostly found in larger‐scale studies when assessed with wearables or actimetry (Mattingly et al., 2021). These tend to show longer sleep in winter and shorter sleep in summer, with effects strongest in children or elderly people, in pre‐industrial societies or without electric light. The last finding supports a confounding role of electric light but its effects in conjunction with photoperiodic changes seems to be unclear. These trends also seem to be challenged by school or work demands, such that one study found that children were reported to sleep longer during school breaks in the summer (Mattingly et al., 2021; Robbins et al., 2020).

The least evidence exists for seasonal variation in sleep micro‐ and macro‐architecture of healthy participants in field studies. Polysomnography (PSG) studies in Antarctica (see review by Pattyn et al. 2018) showed different results from heavily disturbed sleep architecture (Joern et al., 1970) to supposedly normal sleep (Buguet et al., 1987), with sleep disturbances being worse during the Antarctic winter (constant darkness) potentially due to delayed melatonin secretion. However, Antarctica represents an extreme environment for human sleep, thus serving only as an outlier not typical for the living conditions of most people.

The role of changing natural light throughout the year and its interaction with electrical light thus highlights the need of high‐quality longitudinal light exposure data measured with precise and accurate light logging devices to understand its impact on sleep in combination with the changing photoperiod (Spitschan et al., 2022). While the pathways underlying the impact of light on sleep and circadian physiology have been established, with the melanopsin‐containing intrinsically photosensitive retinal ganglion cells (ipRGCs) playing the primary role (Brown, 2020), clear evidence for a link between day‐to‐day light exposure and sleep is lacking. Due to a scarcity of high‐quality light loggers, most research on light exposure has focused on light sensors integrated in actimeters that cannot measure light exposure at eye level. In addition, photopic illuminance, which most prior studies employed, does not accurately reflect the signal available to the ipRGC pathways. Only recently, the physiologically‐relevant melanopic equivalent daylight illuminance (CIE, 2018; Spitschan et al., 2019) has been standardised, allowing to estimate the retinal stimulus available to the ipRGCs.

Overall, there is a gap of high‐resolution and longitudinal sleep architecture and circadian data collected from healthy participants across the year incorporating data on real‐life sleep determinants in addition to light exposure, bedroom temperature conditions, and photoperiod data. Within the Ecology of Human Sleep (EcoSleep) cohort study, we will address this research gap through a longitudinal cohort study with a measurement‐burst design focusing on the sleep variability of 12 healthy young adults across time with a high‐resolution sampling frequency of two EEG nights per month and continuous actimetry recording and light logging for a duration of 12 months. We also include measures for circadian variables and bedroom temperature environment. To our knowledge, this will be the first study to include such a rich set of variables and sensors with high‐resolution sampling over an extensive period.

### 1.1 Research questions

The EcoSleep Project will address the following research questions (RQs):

What is the contribution of individual daytime sleep determinants (including light exposure) on the timing, quality, and architecture of the subsequent sleep episode for the individual?

How does the unique influence of each sleep determinant differ across individuals?

Can photoperiod/time of year predict intra‐individual variation in outcome variables of interest (i.e., sleep‐, circadian‐, non‐parametric circadian‐related variables)?

## 2 METHODS AND ANALYSIS

### 2.1 Study design and timeline

#### 2.1.1 Design, sample size and recruitment

##### Overall design

**FIGURE 1:**
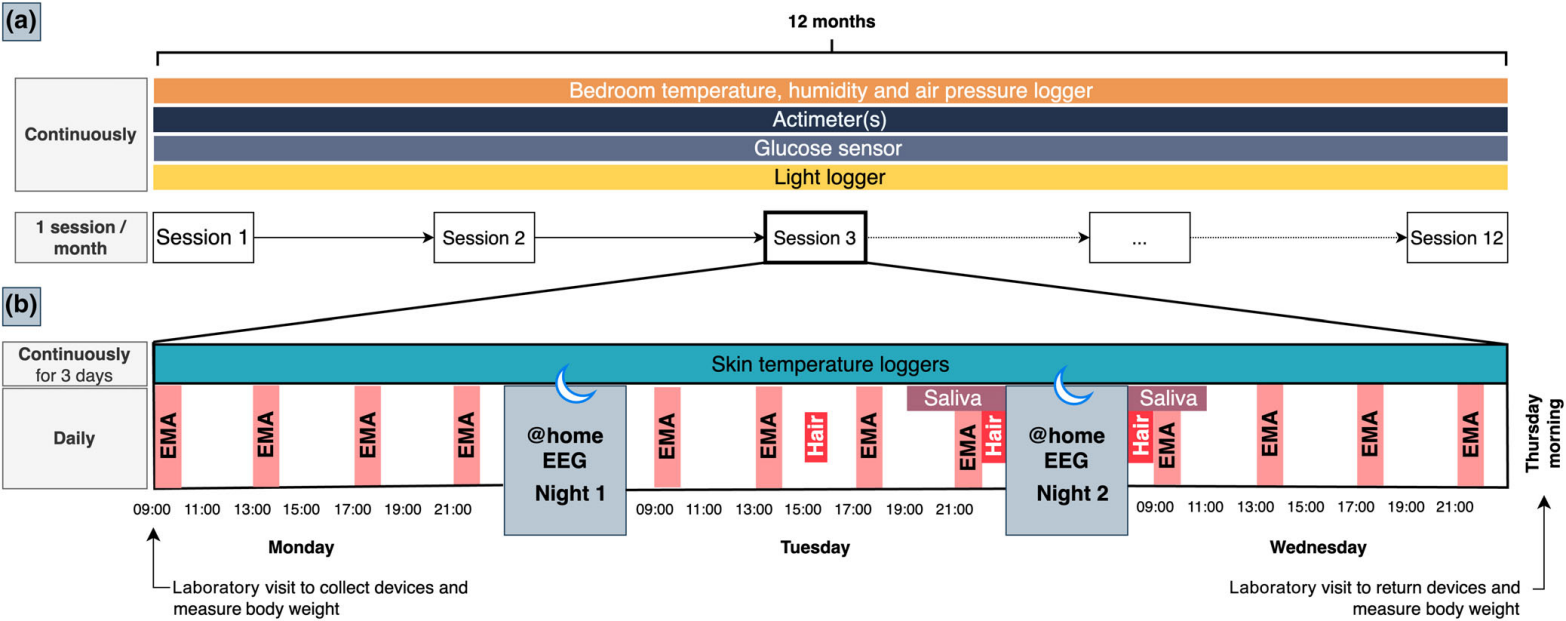
Timeline of the study. Panel (a) shows the entire 12‐month measurement timeline. Panel (b) depicts a session in detail, consisting of 3 consecutive measurement days. EEG, electroencephalography; EMA, ecological momentary assessment.

To understand the fine‐grained relationship between daily life variables on subsequent sleep episodes (RQ1a, b) and to observe variability in sleep architecture over time (RQ2), we will use a measurement burst design (Sliwinski, 2008) focusing on naturally occurring changes during the year (observational study, no intervention). We will examine both short‐term variability in sleep determinants by using four daily ecological assessments and EEG sleep recordings (= bursts) delivered through mobile phones and long‐term changes using repeated bursts over time across the year. To avoid sparse sampling of the phenotype of interest, as is often the case in longitudinal studies (Sliwinski, 2008), the study will run over 12 months with measurement bursts occurring once every four weeks for three consecutive days (see Figure 1 for an overview). We will run the study for 12 months to incorporate seasonal changes. The study will take place in Munich, Germany (48°10′50.4″ N, 11°32′46.5″ E). We previously tested this design in a short‐term feasibility trial and collected quantitative and qualitative feedback. The current protocol was adapted following that feedback.

##### Sample size

We will recruit a total of 12 participants (target 50% female). As this is the first observational study of its kind, there is no principled basis for sample size calculations. We expect a dropout of 30%, leading to an expected final sample of eight participants. The choice of 12 participants is given by resource constraints. We believe that high‐resolution longitudinal data of 12 months duration of eight participants will be informative due to the rich, repeated high‐resolution within‐subjects data.

##### Recruitment

Various recruitment strategies will be used, including fliers and posters placed in and around the Technical University of Munich (TUM), the TUM intranet and via mailing lists. Additionally, participants will be recruited through the TUM Experiment Participant Recruiting System *Sona Systems*, on social media using fliers, and word of mouth.

#### 2.1.2 Inclusion and exclusion criteria

We will apply stringent inclusion and exclusion criteria to control for additional influences that are either well known (e.g., alcohol, age) and/or are very strong determinants of sleep (e.g., sleep or other disorders) but less likely to change daily. To be included in the study, participants need to be physically and mentally healthy adults aged 18–35 years with a score of ≥4 on the 44‐item version of the Big Five Inventory (BFI‐44) subscale for conscientiousness to increase the chance of recruiting participants likely to commit to the 12‐month‐long study. See Table 1 for the inclusion criteria.

**TABLE 1:** Inclusion criteria.

Domain	Criterion	Assessment method
Age	≥18 and ≤35 years of age	Self‐report
Physical health	Good physical health	Self‐report
Mental health	Good mental health	Self‐report
Personality	Highly conscientious, score ≥4	Self‐report (BFI‐44—subscale conscientiousness)

Abbreviation: BFI‐44, 44‐item version of the Big Five Inventory (John et al., 2012).

We will exclude participants who have any known sleep, neurological, metabolic, endocrine, mental, or other physical or mental disorders (including bruxism, as this increases signal noise in EEG recordings). We will also exclude participants who are under‐ or overweight, take medication, persons with extreme chronotypes, smokers, who are not good sleepers, who are on a therapeutic diet (including intermittent fasting), who work rotating or night shifts, are regular video gamers, or sleep in a noisy environment. Pregnant women or persons who are breastfeeding cannot participate in the study. Participants becoming pregnant during the recording period will be retained subsequently. See Table 2 for the exclusion criteria.

**TABLE 2:** Exclusion criteria.

Domain	Criterion	Assessment method
BMI	Underweight or overweight, <18.5, >25 kg/m^2^	Calculation from self‐reported height and weight
Medication use	Any use of medications including hormonal supplements/treatment	Self‐report
Pregnancy or breast feeding	Current pregnancy or currently breast feeding	Self‐report
Bruxism	History of current bruxism	Questionnaire (Pintado et al., 1997)
Smoking	Habitual smoking	Self‐report
Epilepsy	Diagnosis of epilepsy	Self‐report
Health condition	Diagnosis of any neurological, psychological, or psychosomatic conditions	Self‐report
Long COVID	Diagnosis of long COVID	Self‐report
Ocular disease	Diagnosis of any ocular disease or altered colour vision	Self‐report
Substance abuse	Excessive alcohol use, score ≥8	AUDIT
Sleep	Poor sleep quality, score >5	PSQI
Chronotype	Extreme early or late chronotype, MSFsc <1:30 h or >6:00	MCTQ
Gaming behaviour	Extensive gaming behaviour or addiction, score ≥21	IGDS9‐SF
Intermittent fasting or other diet	Current intermittent fasting	Self‐report
Sleep environment	Noise disturbances at sleep environment, score >10	ASE

Abbreviations: ASE, Assessment of Sleep Environment (Grandner et al., 2022); AUDIT, Alcohol Use Disorders Identification Test (Saunders et al., 1993); BMI, body mass index; IGDS9‐SF, nine‐item Internet Gaming Disorder Scale short form (Pontes & Griffiths, 2017); MCTQ, Munich Chronotype Questionnaire (Roenneberg et al., 2003);PSQI, Pittsburgh Sleep Quality Index (Buysse et al., 1989).

#### 2.1.3 Timeline

**FIGURE 2:**
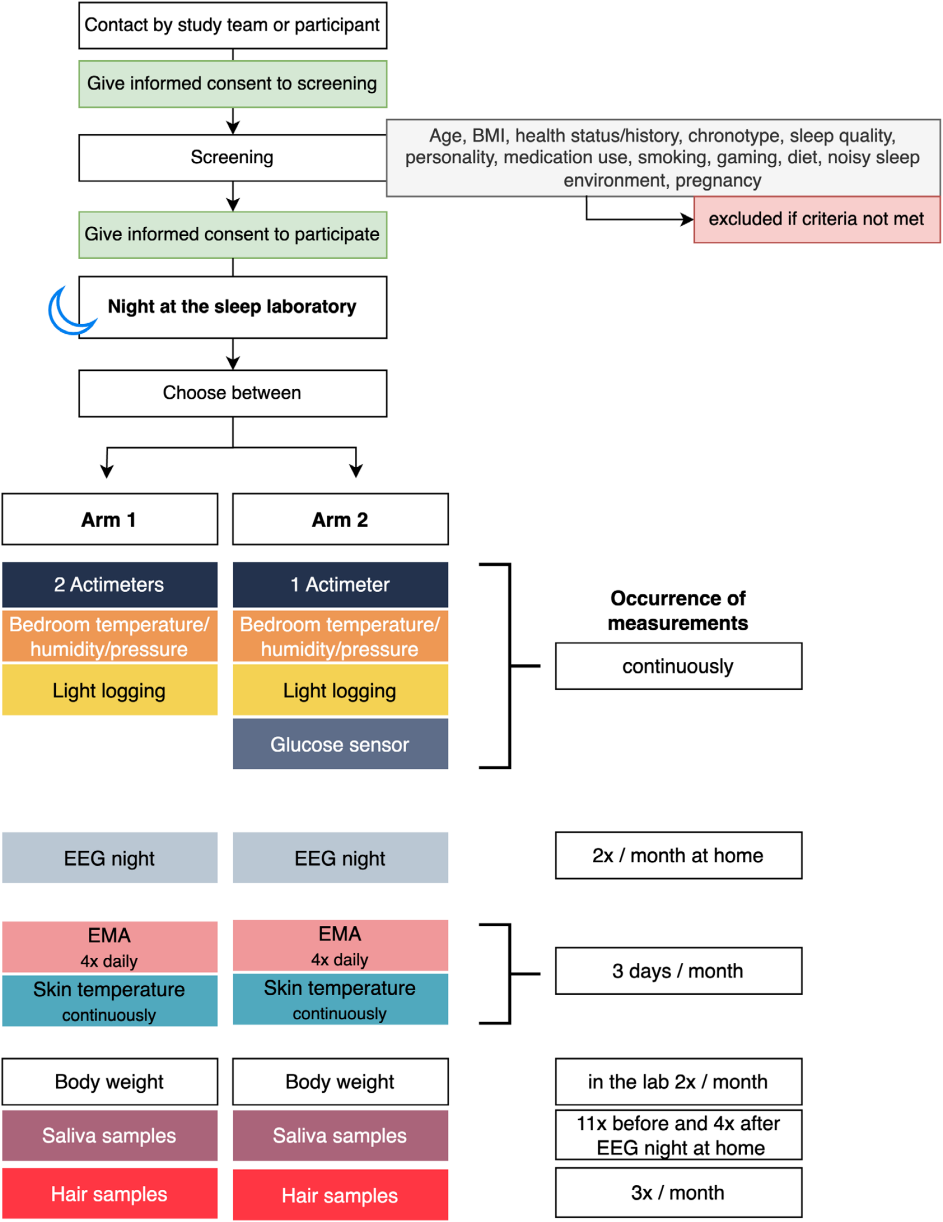
Timeline of onboarding steps and measurements. Participation in *Arm 2* includes the same measurements as *Arm 1* except that participants only wear one type of actimeter and instead additionally measure their glucose levels continuously. BMI, body mass index; EEG, electroencephalography; EMA, ecological momentary assessment.

The entire study will take place over a total of 12 months, with a total of 144 repeated bursts in the entire study sample. Before inclusion, participants will be screened for suitability of participation after consenting to this screening. Upon inclusion, participants give informed consent to participate in the data collection and complete a baseline questionnaire that will be repeated at the end of each month. The baseline questionnaire asks about different determinants of sleep including biological, behavioural, environmental, and personal/socioeconomic determinants. At enrolment, participants can decide between two options of participation (Figure 1 and Figure 2):

##### Arm 1

All participants will continuously wear two actimeters: One wrist‐worn ActTrust2 activity tracker (Condor Instruments; São Paulo, Brazil) and one thigh‐worn Fibion SENS activity tracker (Fibion; Jyväskylä, Finland). Participants will log their light exposure by wearing a ActLumus small light logger (Condor Instruments; São Paulo, Brazil) on their chest in the form of a pendant fixed on a lanyard throughout the study period of 52 weeks. A small temperature logger will be placed on their bedside table that constantly monitors bedroom temperature, humidity, and air pressure. The bedroom environmental data will be enriched with local weather data available from the Meteorological Institute of Ludwig Maximilian University in Munich. Once per month for three consecutive days (so called ‘measurement sessions’), which last from Monday through Wednesday, participants will complete ecological momentary assessments (EMA) four times per day after waking, at ~13:00 local time, at 17:00 local time, and prior to going to bed using the custom‐made *momenTUM* app (https://github.com/momenTUM-research-platform). The EMA questionnaires take about a maximum of 15 min each and ask about sleep timing and quality, physical and mental wellbeing, emotional and mood states, food and drink intake, and any important life events and activities at that time of day. The EMA questionnaires also include the Karolinska Sleepiness Scale (KSS) that probes for current sleepiness levels and ask for first day of the current menstrual cycle. Prior to the EMA questions, participants will also complete a psychomotor vigilance task, which is a simple reaction time task that should take no longer than six min (40 trials). During the two nights of these sessions (i.e., Monday to Tuesday night and Tuesday to Wednesday night), EEG recordings will be obtained at participants’ homes. For this, participants will come to the laboratory on Monday morning to collect their EEG device and return it on Thursday morning. When they are at the laboratory, we will measure their body weight. Throughout the three‐day measurement sessions, participants’ distal–proximal skin temperature gradient will be measured by means of two iButtons (coin‐sized temperature loggers) placed on a distal (lower leg) and proximal (collar bone) body position and fixed with adhesive tape. They will collect these devices when they come to the laboratory. Additionally, participants will collect 12 scalp hair follicle samples at three time points: Tuesday afternoon, Tuesday evening, and Wednesday morning. On Tuesday afternoon, they will also collect saliva samples using Salivettes every 30 min for 11 time points before their habitual bedtime and four time points after waking up on Wednesday morning. Both at‐home hair and saliva collection are guided by a clear instruction sheet discussed with the participants.

##### Arm 2

**FIGURE 3:**
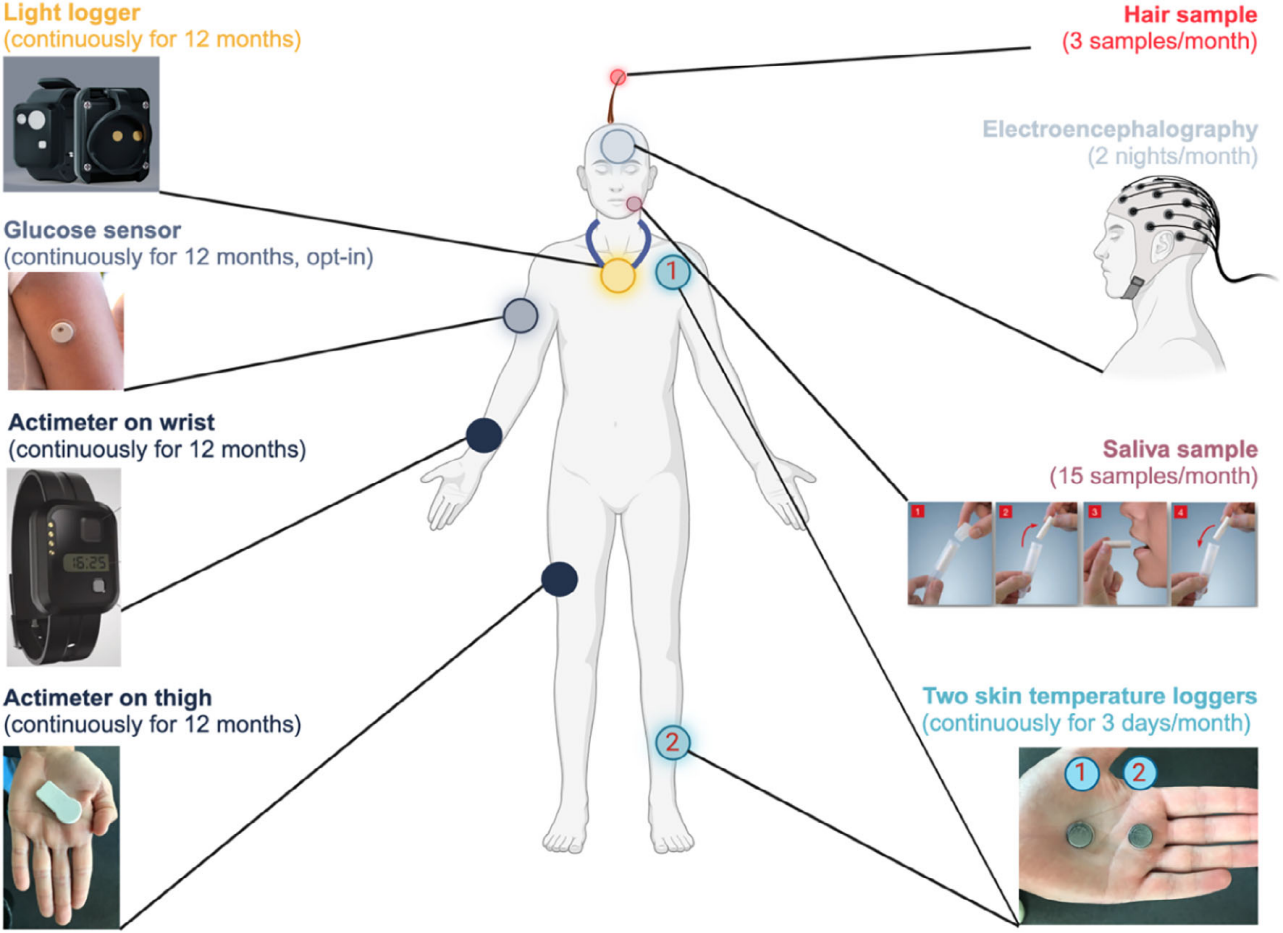
Overview of measurements and sensor and device placements.

A subgroup of six participants only wear one type of actimeter (ActTrust2 from Condor) and continuously monitor their glucose levels in the interstitial fluid using FreeStyle Libre (Abbott GmbH; Wiesbaden, Germany). This glucose sensor is placed at the upper arm and inserted underneath the skin. The sensor can stay attached for up to two weeks after which it has to be replaced. Figure 3 is an overview of body sensors and loggers and their wearing position.

At the end of the study, participants will also complete an evaluation form to evaluate the suitability of the different sensors and the study design. We will interview participants and record their answers to facilitate transcription and content analysis. After transcription audio files will be deleted.

#### 2.1.4 Measurement modalities

All wearable devices, sensors and loggers are shown in detail in Figure 3. Light loggers (ActLumus) from Condor Instruments measure melanopic light exposure at a sample rate of 30 s and are continuously worn around the neck in the form of a pendant for the duration of the study period of 12 months. Glucose sensors (Freestyle Libre) are from Abbott and measure continuous interstitial fluid concentration of glucose for 12 months at a sample rate of 1 min and are placed at the upper arm (opt‐in: participation Arm 2). The actimeters (ActTrust2) are from Condor Instruments sample triaxial acceleration (x, y, z) at a sample rate of 30 s and are worn on the non‐dominant wrist. Activity trackers from Fibion SENS also sample triaxial acceleration at a rate of 30 s and are worn on the thigh. They possibly allow for a better capture of body position and could potentially reduce the amount of non‐wear time. As they are worn on the thigh, they might be preferred by participants who feel uncomfortable wearing a device on their wrist at night during sleep. The ActTrust 2 is worn continuously for 12 months while the Fibion SENS has a shorter battery life and is single use. The Fibion SENS will only be worn for the first month of data collection after which recording with this actimeter will be finished. Hair follicles from scalp hair are sampled by the participant at three time points spaced by ~8 h during monthly measurement sessions (i.e., 3 × 12 months = 36 samples in total) using a kit to estimate circadian phase and amplitude. At each time point, 12 hair follicles are collected from the scalp, placed in a container filled with RNA stabilising solution, and sent to the laboratory immediately after each monthly measurement session. The expression of clock and clock‐controlled genes is analysed using NanoString technology, and circadian phase and amplitude are determined similar to as described in Wittenbrink et al. (2018). EEG recordings will be conducted on two nights during monthly measurements sessions using an ambulatory EEG system (Mentalab Explore, Model EX8M; Munich, Germany) to derive brain activity data for sleep architecture variables. Saliva samples will be taken during the monthly measurement sessions (15 samples every 30 min prior to habitual bedtime and four samples every 30 min the next morning after wake‐up) using Salivettes (Figure 3) to determine circadian phase and cortisol awakening response. Lastly, two temperature sensors in the form of iButtons® are placed at a proximal (collar bone) and distal (shin) position to log skin temperature continuously during the monthly measurement session. EMA will be performed through the custom momenTUM platform, which links an iOS and Android app with server‐side data integration, allowing app‐enabled surveys and other tasks. momenTUM is based on schema (Shatte & Teague, 2019; Shatte & Teague, 2020). Study data will be collected and managed using Research Electronic Data Capture (REDCap) tools hosted at the Technical University of Munich (Harris et al., 2009; Harris et al., 2019). REDCap is a secure, web‐based software platform designed to support data capture for research studies, providing: (i) an intuitive interface for validated data capture; (ii) audit trails for tracking data manipulation and export procedures; (iii) automated export procedures for seamless data downloads to common statistical packages; and (iv) procedures for data integration and interoperability with external sources.

### 2.2 Data analysis plan

The study includes several variables, including *sleep determinants* and sleep‐ and circadian‐related *outcome variable*s.

#### 2.2.1 Sleep determinants

In RQ1a, b, we aim to understand which individual sleep determinants (= predictors of sleep) prior to the sleep episode (i.e., factors on day 1; listed in Table 3) influence our main sleep and circadian outcome variables as listed in detail in Table 4. To achieve this, we will collect biological, behavioural, (physical) environment and personal/socioeconomic (Table 3) sleep determinants as outlined by the Public Health Classifications Project for Determinants of Health (Philippens et al., 2022). Please note that we decided against collecting data on ethnicity due to the small sample size and complex categories of ethnicity that are difficult to assess in the German population. For RQ2, we are mainly interested in observing changes in our primary outcome measures across time of year and within an individual.

**TABLE 3:** Overview of included sleep determinants and time of assessment.

Biological determinants	Behavioural determinants	Environmental determinants	Personal and socioeconomic determinants	Assessed at
Age^a^	Alcohol and caffeine intake^a^	Noise^a^	Personality^a^ and attachment style	**Baseline**
Sex and gender	Gaming behaviour^a^	Natural disasters	Ethnicity^b^	
Chronotype^a^	Intermittent fasting^a^		Socioeconomic status	
	Exercise		Psychological disposition	**EMA** (once per month 4× daily, for 3 consecutive days)
	Meditational physical activities	Light exposure behaviour	Mood	
			Psycho‐social stress	
	Music listening		Social relations	
	Physical activity/Sedentary behaviour	Bedroom temperature, humidity, air pressure		**Continuously**
		Light exposure		

*Note*: Natural disasters, major political events and other events of public life will be recorded by the study team if relevant during the recording period.

Abbreviation: EMA, ecological momentary assessment.

^a^ Part of the exclusion criteria to control for this determinant.

^b^ Not assessed due to small sample size and inadequate ethnic categories for Germany.

**TABLE 4:** Overview of primary outcome variables.

Outcome	Abbreviation	Format	Computation with software or formula	Assessed with	Assessed at
**Sleep variables**					
**Sleep onset**	Son	Clock time (local time)	–	Sleep diary	EMA
			ActStudio (Condor Instruments)	Actimetry	Continuously (30‐s resolution)
**Sleep offset**	Soff	Clock time (local time)	–	Sleep diary	EMA
			ActStudio (Condor Instruments)	Actimetry	Continuously (30‐s resolution)
**Sleep duration**	Sdur	Numeric (min)	–	Sleep diary	EMA
			ActStudio (Condor Instruments)	Actimetry	Continuously (30‐s resolution)
**Midsleep**	MS	Clock time (local time)	–	Sleep diary	EMA
			ActStudio (Condor Instruments)	Actimetry	Continuously (30‐s resolution)
**Absolute social jetlag**	SJL	Clock time (local time)	|Midsleep on free days—midsleep on weekdays|	Actimetry	Continuously (30‐s resolution)
**Subjective sleep quality**	SQ	11‐point Likert scale	–	Sleep diary	EMA
**Wake after sleep onset** Objective sleep quality approximation	WASO	Numeric (min)	Total time spent awake after sleep onset YASA algorithm (Vallat & Walker, 2021)	EEG	2 nights/month
**Sleep architecture** % and absolute duration of sleep stages (awake, N1, N2, N3, REM)	Awake N1 N2 N3 REM	%	(Time spent in sleep stage/total sleep time) × 100 YASA algorithm (Vallat & Walker, 2021)	EEG	2 nights/month
**Sleep onset latency**	SOL	Numeric (min)	Time from lights out to first epoch of any sleep YASA algorithm (Vallat & Walker, 2021)	EEG	2 nights/month
**REM latency**	lat_REM	Numeric (min)	Time from start of recording to first REM sleep episode YASA algorithm (Vallat & Walker, 2021)	EEG	2 nights/month
**Sleep efficiency**	SE	%	(Total sleep time/time in bed) × 100 YASA algorithm (Vallat & Walker, 2021)	EEG	2 nights/month
**Total sleep time**	TST	Numeric (min)	Sum of time spent in all sleep stages YASA algorithm (Vallat & Walker, 2021)	EEG	2 nights/month
**Circadian variables**					
**Amplitude**	A	Numeric	Maximum‐mesor (of best fitting cosine function)	Actimetry	Continuously (30s resolution)
				Scalp hair follicle (from mRNA) (Wittenbrink et al., 2018)	1×/month (three samples necessary)
**Phase**	ϕ	Clock time (local time)	Time of highest activity values	Actimetry	Continuously (30‐s resolution)
				Scalp hair follicle (from mRNA) (Wittenbrink et al., 2018)	1×/month (three samples necessary)
**Habitual melatonin onset**	mel_on	Clock time (local time) and AUC	>3 pg/mL and 2 SD above the mean of three baseline values (Danilenko et al., 2014) and hockey‐stick algorithm (Danilenko et al., 2014)	Saliva samples	1×/month (15 samples necessary)
**Habitual melatonin offset**	mel_off	Clock time (local time) and AUC	<3 pg/mL and hockey‐stick algorithm (Danilenko et al., 2014)	Saliva samples	1×/month (15 samples necessary)
**Cortisol awakening response**	CAR	Mean cortisol increase after wake and AUC, AUC_G_ and AUC_I_ (Pruessner et al., 2003; Stalder et al., 2016)	μg/dL	Saliva samples	1×/month (four samples necessary)
**Non‐parametric circadian variables**					
**Intradaily variability** = amount of fragmentation of the rhythm	IV	Numeric	pyActigraphy (Hammad et al., 2021) / ActStudio (Condor Instruments)	Actimetry	Continuously (30‐s resolution)
**Interdaily stability** = relative strength of the circadian rhythm	IS	Numeric	pyActigraphy (Hammad et al., 2021) / ActStudio (Condor Instruments)	Actimetry	Continuously (30‐s resolution)
**Nocturnal activity**= average activity during the least active 5‐h period	L5	Numeric	pyActigraphy (Hammad et al., 2021) / ActStudio (Condor Instruments)	Actimetry	Continuously (30‐s resolution)
**Daytime activity** = average activity during the most active 10‐h period	M10	Numeric	pyActigraphy (Hammad et al., 2021) / ActStudio (Condor Instruments)	Actimetry	Continuously (30‐s resolution)

Abbreviations: AUC, area under the curve; AUC_G_, Area under the curve with respect to ground; AUC_I_, Area under the curve with respect to increase; EEG, electroencephalography; EMA, ecological momentary assessment; SD, standard deviation; WASO, wake after sleep onset; YASA, Yet Another Spindle Algorithm (Benedetti et al., 2023).

#### 2.2.2 Primary outcome variables

Our primary outcome variables include sleep outcomes, circadian‐related outcomes, such as phase and amplitude, as well as cortisol awakening response as listed in Table 4. Sleep outcomes include: sleep onset, offset, duration and midsleep; social jetlag; subjective sleep quality; wake after sleep onset (WASO); sleep architecture including percentage and duration of sleep stages (N1–N3, REM); sleep onset latency and REM‐sleep latency; sleep efficiency; and total sleep time. Circadian variables include: amplitude and phase, habitual salivary melatonin onset and offset, and timing and magnitude of the cortisol awakening response. Non‐parametric variables of circadian rhythms include intradaily variability, interdaily stability, nocturnal activity (average activity during the least active 5‐h period) [L5], and daytime activity (average activity during the most active 10‐h period) [M10]. For definitions and calculation see Table 4.

#### 2.2.3 Data pre‐processing

Within three months after starting data collection a data pre‐processing and analysis plan will be formulated in response to newly acquired knowledge of data quality and participant adherence to the study procedures. This data pre‐processing plan will include descriptions of strategies applied for handling different types of missing and/or noisy data. We will explore whether existing smoothing techniques that address outlier and missing data handling (e.g., nonlinear smoothing of data with random gaps and outliers [DRAGO]; Obeid et al., 2021) and imputation methods such as median imputation (e.g., as described by Weed et al., 2022) can be exploited for our data, as well and how different amounts and timing of missing data influences our outcome metrics of interest. The data pre‐processing and analysis plan will be pre‐registered and deviations from the pre‐registration will be described in future articles.

Light exposure can be quantified in various metrics which are listed in the Supplementary Table S1. Quantification of the remaining sleep determinants as listed in Table 3 (e.g., physical activity, mood, etc.) will also be done. Pre‐processing steps will include determining data quality cut‐offs, adequate (if necessary) aggregation levels of both sleep determinants and outcome variables, and deciding on exclusion/imputation methods (e.g., trimming versus winsorizing) and on detrending the data. Detrending is necessary if dynamic models (e.g., cross‐lagged models or continuous time models) will be employed. Diagnostic tools to ensure model residuals are white noise (random errors) could include residual plots and QQ plots. We will also test and account for collinearity of variables (e.g., light exposure might not be independent of time of year) within all models using the variance inflation factor (VIF).

If data collection falls within daylight savings time (DST) transitions, data will be analysed both in relation to local time (clock time) and photoperiod (sun time). Data from transitions periods might be disregarded or analysed separately given that DST changes have shown to influence sleep (as for example reviewed by Harrison, 2013). Categorised season is defined following standard meteorological definitions for the Northern hemisphere as follows: spring (from March 1 to May 31), summer (from June 1 to August 31), autumn (from September 1 to November 30), winter (from December 1 to February 28). Seasonal and time of year information can be entered as a categorical variable (winter, spring, summer, autumn), or numeric (photoperiod length or time of year in day number). This has to be further explored based on data quality and structure.

Data will be processed using software packages provided by the manufacturers of the devices and open access Python or R based packages. These include *ActStudio* (version 1.0.23) for ActTrust (actimeter) and ActLumus (light logger) from Condor Instruments, and Fibion SENS App (version 3.7.0–240111) for the Fibion device (actimeter). Future versions for these software packages may be used and will be named in future publications on these data.

#### 2.2.4 Statistical analyses

Statistical analyses will be using R (version 2023‐10‐31), R Studio (version 2023.12.1+402) and python (version 3.12.1) or future version if available at the time of analysis. Particular R packages that are planned to be used, among others, include *ctsem* (Driver et al., 2017; Driver & Voelkle, 2018) or *forecast* (Hyndman & Khandakar, 2008) for continuous time dynamic modelling and autoregressive models, *lme4* (Bates et al., 2015) for linear mixed models, *lavaan* for structural equational modelling (Rosseel, 2012), *car* (Fox & Weisberg, 2011) for testing collinearity, *effects* (Fox & Weisberg, 2018) for tabular and graphical effects display, *ggplot2* (Wickham, 2016) for visualising data, *tidyverse* (Wickham et al., 2019) for data wrangling, and *LightLogR* (Zauner & Spitschan, 2023) for processing and calculating light data and variables. Python packages include *pyActigraphy* (Hammad et al., 2021) for actimetry data analysis, and Yet Another Spindle Algorithm (*YASA*; Benedetti et al., 2023) algorithm for sleep determining sleep stages from EEG data.

##### Descriptive statistics

Standard descriptive statistics, e.g., mean, standard deviation, minimum and maximum, skewness and curtosis will be reported for all metric variables, both within‐persons and between persons. If normality is violated or for non‐numeric variables, we will use robust descriptive measures (median, interquartile range). Frequencies and percentages will be reported for categorical variables. Pearson correlations will be used for computing numerical correlations, Spearman's rank correlations will be used for ordinal data. Correlation matrices will be reported on a group and individual level to determine correlation of variables. Trends in time series data throughout the year will be graphically represented for key outcomes of interest.

##### Modelling

For RQ1a (i.e., the influence of sleep determinants on sleep outcome variables and vice versa) and if data quality allows (see section on Data pre‐processing), we plan to use descriptive summary statistics and potentially correlation matrices for group and individual analyses as described above (see Descriptive statistics). For modelling, our focus will lie on both discrete‐time and continuous‐time dynamic modelling methods. These will be utilised to delve into and examine the temporal interplay between sleep outcome variables, as listed in Table 4, and sleep determinants, as outlined in Table 3. This approach is aimed at a comprehensive examination of the dynamic relationships between these key sleep‐related factors. Autocorrelation function and plots will be employed to examine autocorrelation at different lags and explore trajectories to decide on a set of potential models that are subsequently tested against each other. Model fits will be assessed using chi‐square tests (with a significance level of *α* = 0.05 along with descriptive model fit indices, such as the root mean square error of approximation and the comparative fit index), provided they are feasible to calculate.

To address RQ1b (i.e., the unique influence of each sleep determinant per person), we will use models with between‐person random cross‐lagged effects, as implemented in Bayesian frameworks, e.g., as described in Driver & Voelkle, 2018.

For RQ2 (i.e., seasonal variation in outcome variables), we will mainly use descriptive summary statistics to describe intra‐ and inter‐individual variability of our outcome variables (as outlined in Table 4) across time of year/season/photoperiod including time‐series plots. In addition, we consider time series and trend analyses as for RQ1a, b. We will also employ regression‐based analyses, including linear mixed modelling, to predict outcome variables by time of year/photoperiod. Note that we generally expect the greatest differences in outcome variables between summer (long photoperiod) and winter (short photoperiod).

###### Model comparisons

For comparing models, we intend to use likelihood ratio tests (with a significance level of *α* = 0.05) for nested models, information criteria, and/or Bayes factors.

###### Expected problems and limitations

Given the anticipated small sample size of individuals, conducting between‐person statistical analyses may be limited and may not be feasible depending on data quality. In such instances, greater emphasis will be placed on descriptive and individual‐level *n‐of‐1* analyses. In general, further explorative analyses are likely to be conducted depending on data quality and availability.

## 3 ETHICS AND DATA MANAGEMENT

### 3.1 Ethical approval

A feasibility trial of this study was reviewed and approved by the TUM Ethics Committee on November 14, 2022 under #2022‐578‐S‐KH. The current protocol includes major changes to the feasibility trial, which were reviewed and positively evaluated by the TUM Ethics Committee on February 1, 2024 under #2023‐653‐S‐SB.

### 3.2 Data collection and management

Data will be collected by qualified study staff only. Data will be collected pseudonymised using a study identifier (ID) for each participant. Only authorised study personal will have access to a list de‐identifying participant ID and study ID of the respective person. All data that we can assess independently will be stored securely on an internal TUM server. We adhere to European Union General Data Protection Regulations.

Data from Fibion SENS (actimeter data) transmitted via Bluetooth and stored on their server (server location in Frankfurt, Germany), are processed, and provided for download on a webpage to which only authorised personnel have access to. Glucose levels in interstitial fluid will be read using a reader or smartphone application from Freestyle Libre Abbott and stored on their server (AWS). We will ensure additional pseudonymisation to meet data security concerns (different study ID to their original study ID) in this case.

## 4 DISSEMINATION

Our findings will be presented at regional, national, and international scientific conferences and workshops and submitted for publication in peer‐reviewed journals. We also adhere to open science principles, including this open protocol. We will publish code under the Massachusetts Institute of Technology (MIT) Licence, and materials and data under the Creative Commons (CC‐BY) licence on GitHub (https://tscnlab.org/). The study's results could inform future interventions using eHealth and other digital‐health methods.

We also plan to present results of the study in non‐scientific contexts to inform the public through outreach events and public engagement formats. Study participants will receive a summary of their data in simplified language to help them understand their own (sleep) data, including sleep hygiene behaviour, light exposure and chronotype.

## 5 STRENGTHS AND LIMITATIONS

- This study investigates human sleep in the natural environment across 12 months incorporating multi‐domain sleep determinants to understand their combined contribution to the subsequent sleep episode.
- The study integrates novel and state‐of‐the art data collection methods, including wearable at‐home EEG, continuous glucose measurement (CGM) and personalised light logging, as well as hair follicle‐derived circadian amplitude and phase.
- The study focuses on longitudinal and high‐resolution intra‐individual data (*N* = 12) going beyond sparse resolution. Assessments include home‐based EEG recordings twice per month, monthly circadian phase and amplitude assessment, 3‐days of four daily ecological momentary assessment per month, and continuous actimetry, continuous light logging and continuous bedroom temperature/humidity/air pressure monitoring.
- Due to the lack of experimental manipulations, drawing direct causal inferences from the data will not be possible.
- The participant burden to generate the within‐subject data is high due to the intensive sampling and long participation duration.

## AUTHOR CONTRIBUTIONS

**Anna M. Biller:** Conceptualiation; methodology; data curation; writing – review and editing; writing – original draft; resources; project administration; visualization; funding acquisition; supervision. **Nayab Fatima:** Data curation; resources; writing – original draft; writing – review and editing. **Chrysanth Hamberger:** Writing – review and editing; data curation; methodology; resources; visualization. **Laura Hainke:** Methodology; resources; writing – review and editing. **Verena Plankl:** Resources; writing – review and editing. **Amna Nadeem:** Writing – review and editing. **Achim Kramer:** Writing – review and editing; resources; methodology. **Martin Hecht:** Writing – review and editing; methodology. **Manuel Spitschan:** Conceptualization; funding acquisition; writing – review and editing; writing – original draft; resources; supervision; methodology; project administration.

## FUNDING INFORMATION

This work is supported by the TUM Seed Fund (awarded to Manuel Spitschan), the Max Planck Society (Max Planck Free‐Floating Research Group to Manuel Spitschan) and the Deutsche Gesellschaft für Schlafforschung und Schlafmedizin e. V. (award to Anna M. Biller).

## CONFLICT OF INTEREST STATEMENT

All authors have completed the ICMJE uniform disclosure form at http://www.icmje.org/disclosure-of-interest/ and declare the following: **Anna M. Biller** received financial support from the German Sleep Society—Deutsche Gesellschaft für Schlafforschung und Schlafmedizin e. V. for the submitted work; **Laura Hainke** has previously been employed at Mentalab GmbH (prior to the submitted work) but is no longer affiliated with the company; **Manuel Spitschan** received financial support from the TUM Seed Fund and the Max Planck Society (Max Planck Free‐Floating Research Group); **Verena Plankl** is currently employed at the sleep laboratory of the Klinikum Rechts der Isar, Munich, Germany in which the laboratory‐based recording are currently planned to take place; **Achim Kramer** is a shareholder of BodyClock Technologies GmbH and receives partial licencing fees from BodyClock; **all other authors** declare no financial relationships with any organisation that might have an interest in the submitted work in the previous three years; **all authors** declare no other relationships or activities that could appear to have influenced the submitted work.

## Supporting information


**Data S1.** Supporting Information.

## Data Availability

Data sharing is not applicable to this protocol article as no new data were created or analyzed in this study. However, we will publish code under the MIT License, and materials and data under the Creative Commons (CC‐BY) license on GitHub (https://github.com/tscnlab) once the data collection is complete.
