# Supplementary material for: The Ecology of Human Sleep (EcoSleep) Cohort Study: Protocol for a longitudinal repeated measurement burst design study to assess the relationship between sleep determinants and outcomes under real‐world conditions across time of year
Source: J Sleep Res. 2024 Jul 22;34(2):e14225. doi: 10.1111/jsr.14225 (PMC11911042; doi:10.1111/jsr.14225)
Supplement: Supplementary file 1 — Data S1. Supporting Information. [file JSR-34-e14225-s001.docx]

# Supplementary Material

**Title:**

“The Ecology of Human Sleep (EcoSleep) Project: Protocol for a longitudinal cohort repeated-measurement-burst study to assess the relationship between sleep determinants and sleep outcomes under real-world conditions across time of year”

**Authors:**

Anna M Biller ^[0000-0002-3673-8838]^

Nayab Fatima ^[0009-0006-5856-7642]^

Chrysanth Hamberger ^[0009-0005-1082-3451^

Laura Hainke ^[0000-0002-8348-5554]^

Verena Plankl ^[0009-0006-2030-7052]^

Amna Nadeem ^[0009-0000-4720-6792]^

Achim Kramer ^[0000-0001-9671-6078]^

Martin Hecht ^[0000-0002-5158-4911]^

Manuel Spitschan ^[0000-0002-8572-9268]^

**Contact details corresponding author:**

Dr. Anna M Biller

Email: [anna.biller@tum.de](mailto:anna.biller@tum.de)

| **Light exposure variables** | | | | | |
| --- | --- | --- | --- | --- | --- |
| **Nocturnal light exposure**  = average light exposure during the last active 5-h period | L5_light | Numeric | LightLogR [1] | ActLumus | Continuously for 3 days/month  (30 s resolution) |
| **Daytime light exposure**  = average light exposure during the most active 10-h period | L10_light | Numeric | LightLogR [1] | ActLumus | Continuously for 3 days/month  (30 s resolution) |
| **Time above threshold**  (>XXX lux) | TAT_XXX | Numeric  (minutes) | LightLogR [1] | ActLumus | Continuously for 3 days/month  (30 s resolution) |
| **Mean light above threshold** | MLit^XXX^ | Numeric  (minutes since midnight) | Average clock time of all aggregated data points above XXX lux  LightLogR [1] | ActLumus | Continuously for 3 days/month  (30 s resolution) |
| **Melanopic EDI** | mel_EDI | Numeric  (lux) | LightLogR [1] | ActLumus | Continuously for 3 days/month  (30 s resolution) |

**Supplementary Table 1.** **Overview of light exposure quantifications.** XXX is a placeholder for the desired threshold, e.g., 500 lux.

# References Supplementary Material

[1] Zauner, Johannes, Spitschan M. LightLogR: Working With Wearable Light Logger Data. R Package 2023. https://github.com/tscnlab/LightLogR (accessed January 17, 2023).
